# Anatomo-functional basis of emotional and motor resonance elicited by facial expressions

**DOI:** 10.1093/brain/awae050

**Published:** 2024-02-14

**Authors:** Maria Del Vecchio, Pietro Avanzini, Marzio Gerbella, Sara Costa, Flavia Maria Zauli, Piergiorgio d’Orio, Elena Focacci, Ivana Sartori, Fausto Caruana

**Affiliations:** Institute of Neuroscience, National Research Council of Italy (CNR), 43125 Parma, Italy; Institute of Neuroscience, National Research Council of Italy (CNR), 43125 Parma, Italy; Department of Medicine and Surgery, University of Parma, 43125 Parma, Italy; Department of Medicine and Surgery, University of Parma, 43125 Parma, Italy; ‘Claudio Munari’ Epilepsy Surgery Center, ASST GOM Niguarda, 20142 Milan, Italy; ‘Claudio Munari’ Epilepsy Surgery Center, ASST GOM Niguarda, 20142 Milan, Italy; Department of Medicine and Surgery, University of Parma, 43125 Parma, Italy; ‘Claudio Munari’ Epilepsy Surgery Center, ASST GOM Niguarda, 20142 Milan, Italy; Institute of Neuroscience, National Research Council of Italy (CNR), 43125 Parma, Italy

**Keywords:** stereo-electroencephalography, anterior insula, Rolandic operculum, mirror neurons, emotional expressions, facial mimicry

## Abstract

Simulation theories predict that the observation of other’s expressions modulates neural activity in the same centres controlling their production. This hypothesis has been developed by two models, postulating that the visual input is directly projected either to the motor system for action recognition (motor resonance) or to emotional/interoceptive regions for emotional contagion and social synchronization (emotional resonance). Here we investigated the role of frontal/insular regions in the processing of observed emotional expressions by combining intracranial recording, electrical stimulation and effective connectivity.

First, we intracranially recorded from prefrontal, premotor or anterior insular regions of 44 patients during the passive observation of emotional expressions, finding widespread modulations in prefrontal/insular regions (anterior cingulate cortex, anterior insula, orbitofrontal cortex and inferior frontal gyrus) and motor territories (Rolandic operculum and inferior frontal junction). Subsequently, we electrically stimulated the activated sites, finding that (i) in the anterior cingulate cortex and anterior insula, the stimulation elicited emotional/interoceptive responses, as predicted by the ‘emotional resonance model’; (ii) in the Rolandic operculum it evoked face/mouth sensorimotor responses, in line with the ‘motor resonance’ model; and (iii) all other regions were unresponsive or revealed functions unrelated to the processing of facial expressions. Finally, we traced the effective connectivity to sketch a network-level description of these regions, finding that the anterior cingulate cortex and the anterior insula are reciprocally interconnected while the Rolandic operculum is part of the parieto-frontal circuits and poorly connected with the former.

These results support the hypothesis that the pathways hypothesized by the ‘emotional resonance’ and the ‘motor resonance’ models work in parallel, differing in terms of spatio-temporal fingerprints, reactivity to electrical stimulation and connectivity patterns.

## Teaser

Del Vecchio *et al.* use stereo-EEG to shed light on the role of the frontal lobes in the processing of observed emotional expressions. They test the two predominant theories of face processing by combining intracranial recording, electrical stimulation, and effective connectivity tracing in surgical patients.

## Introduction

The observation of emotional facial expressions triggers a cascade of neural activations beyond the occipito-temporal face network,^1,2^ involving multiple frontal areas. Over the years, ‘simulation theories’ tried to account for these activations arguing that other’s expressions resonate in the same regions controlling their production, and that such resonance is likely mediated by a mirror-neuron system for facial expressions, similar to the one classically described for hand actions.^3-8^ This hypothesis is substantiated, at the behavioural level, by the automatic tendency to respond to emotional expressions with unobservable or mild facial changes.^9,10^

At the system neuroscience level, this perspective has been modelled by two simulationist models, which postulated that the matching between observed and executed emotional expressions occurred either within motor/premotor areas^7,11^ (motor resonance) or emotional/interoceptive regions^12,13^ (emotional resonance). Although similar at first glance, the hypothesis that the observation/execution matching takes place in a motor rather than emotional/interoceptive territories has repercussions on its functional contribution to cognitive processes, spanning from action recognition through its covert replication^8^ (motor resonance) to social synchronization and emotional contagion^14^ (emotional resonance). Moreover, even assuming that both systems are at work,^15^ it would remain to be clarified their possible connections or whether the two systems are hierarchically independent.

To shed light on the role of frontal/insular regions in the processing of observed emotional expressions, and to investigate the motor and emotional resonance models of face processing, the present study takes a three-pronged approach that relies on three different tools provided by invasive investigations in drug-resistant epileptic patients, namely intracranial recording, high-frequency electrical stimulation and effective connectivity.

First, we performed direct measurements of the neural activity elicited by the passive observation of static emotional (smiling and fearful) expressions, compared to neutral ones, by recording intracranial event-related potentials (iERPs) from the entire frontal and insular cortices in 44 subjects (5663 recording sites). Such a high sampling allowed us to obtain unique spatio-temporal information on the processing of faces in prefrontal, premotor and anterior insular territories. Despite motor/emotional resonance and facial mimicry may be facilitated by the observation of dynamic (rather the static) expressions,^16,17^ and frontal activations are more commonly found during explicit emotion recognition (rather than passive observation) tasks,^18^ the passive observation of static expressions allowed us to implement a highly-controlled and time-locked experiment and, at the same time, to use the same methodological choices adopted in the classical studies of facial mimicry.^9^

Once the responding sites of interest were identified, we causally explored the functional relevance of each site by reviewing the behavioural and subjective effects elicited by their high-frequency electrical stimulation (HF-ES). Such information is crucial to establish whether the visual information on others’ expressions is projected to a territory involved in face motor functions (as predicted by ‘motor resonance’), to emotional/interoceptive territories (as predicted by ‘emotional resonance’), or to regions unresponsive to HF-ES and likely unrelated to both functions. In the third and last step, we capitalized on the possibility of studying the cortico-cortical evoked potentials (CCEPs) induced by single-pulse electrical stimulation (SPES) to trace the effective connectivity of the responding regions and to provide a network-level description of the involved regions.

Our results make a case for a new hypothesis that the observation of emotional expressions leads to the concomitant modulation of two independent streams of information, differing in terms of spatio-temporal fingerprints, reactivity to electrical stimulation and connectivity patterns.

## Materials and methods

### Participants

The study involves a cohort of 44 patients affected by drug-resistant epilepsy undergoing stereo-electroencephalographic (SEEG) implantation of intracerebral electrodes as part of the presurgical evaluation^19,20^ at the ‘Claudio Munari’ Center for Epilepsy Surgery, Niguarda Hospital, Milan, Italy, between May 2015 and April 2021. Investigations included left hemisphere (*n* = 17), right hemisphere (*n* = 20), and bilateral (*n* = 7) implantations. All these patients were recruited for a perceptual task, consisting of passive observation of static emotional expressions (see later).

The selection of patients has been submitted to a series of stringent precautionary measures. The inclusion/exclusion criteria are the same employed in our previous studies.^21^ The present study received the approval of the Ethical Committee of Niguarda Hospital (ID 939-12.12.2013). Patients were fully informed of the recording procedures and signed informed consent to participate in the study, according to the Declaration of Helsinki.^22^

### Electrode implantation and anatomical reconstruction

SEEG electrodes were implanted only for clinical purposes. The investigated hemispheres, the location and the number of explored sites were based on hypotheses about the seizure onset zone (SOZ) derived clinical history and examination, non-invasive long-term video-EEG monitoring, and neuroimaging.^20,23^ Each subject underwent brain MRI (Achieva 1.5 T, Philips Healthcare) and CT (O-arm 1000 system, Medtronic) to acquire appropriate sequences for SEEG planning. The duration of the SEEG investigation was based only on clinical needs. Placement of intracerebral electrodes was performed under general anaesthesia by means of a robotized passive tool-holder (Neuromate, Renishaw Mayfield SA). A variable number of platinum–iridium semi-flexible multi-lead intracerebral electrodes, with a diameter of 0.8 mm, a lead length of 2 mm, an inter-leads distance of 1.5 mm and a maximum of 18 leads per electrode (Microdeep intracerebral electrodes, D08, Dixi Medical) were placed and fixed. After implantation, a fine cone-beam CT data set was acquired by using the O-arm and co-registered with the T1-weighted 3D MR image to verify the actual position of the electrodes. The anatomical reconstruction procedure has been described in previous studies from our group.^24^

### Intracranial recordings: experimental paradigm

**Figure 1:**
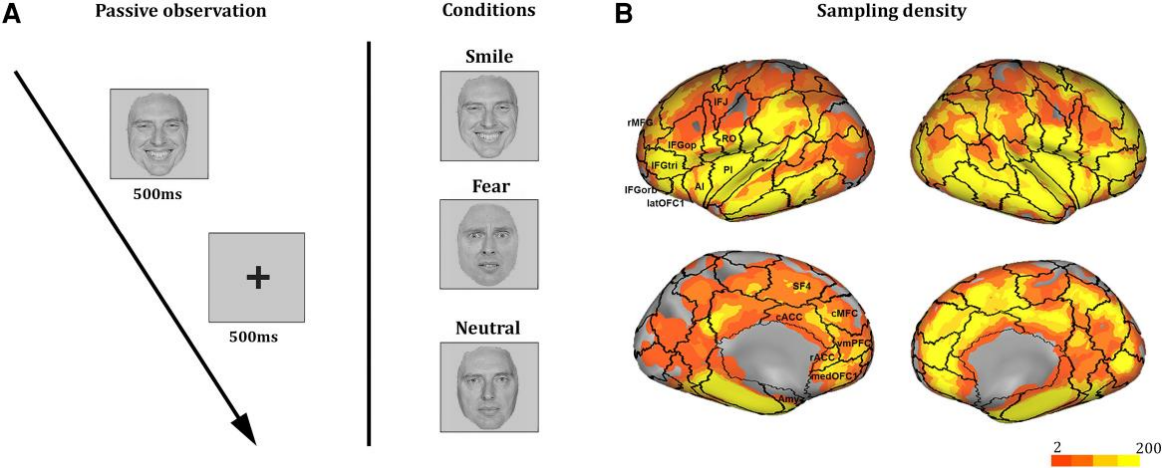
**Experimental stimuli and sampling density**. (**A**) The experimental paradigm was composed of static images depicting human faces and followed by a fixation cross. Facial expressions include positive, negative and neutral expressions. (**B**) The site sampling density is shown on the inflated surface of fs_LR brain template. The colour scale indicates the number of leads within a disk of 2 cm of radius and centred on each node of the mesh. Black boarders indicate are based on the Lausanne2008 (resolution 60) parcellation. The Lausanne2008 (resolution 60) parcellation schema is illustrated on the inflated surface of the FS_LR brain template. AI = anterior insula; Amy = amygdala; cACC = caudal anterior cingulate cortex; cMFC = caudal middle frontal gyrus; IFG = inferior frontal gyrus; IFGop = IFG pars opercularis; IFGorb = IFG pars orbitalis; IFGtri = IFG pars triangularis; IFJ = inferior frontal junction; Lat OFC1 = lateral orbitofrontal cortex 1; med OFC1 = medial orbitofrontal cortex; PI = posterior insula; rMFG = rostral middle frontal gyrus; rACC = rostral anterior cingulate cortex; RO = Rolandic operculum; SF4 = superior frontal 4; vmPFC = ventromedial prefrontal cortex.

During the study, the patient sat approximately 60 cm away from the laptop display where the stimuli were presented. Recordings were obtained in a dimly light, quiet room. Stimuli consisted of static grey-scale images of emotionally expressive faces (five women and three men, adults and of the same ethnicity as the participants) depicting two emotional expressions, i.e. smiling and fearful, and a neutral one. Images were taken from Ekman and Friesen’s^25^ set of pictures of facial affect, i.e. the same stimuli used by Dimberg^9^ to elicit facial mimicry. While there is a broad consensus that the emergence of facial mimicry is facilitated by the observation of dynamic expressions, static expressions are processed more accurately and quickly than dynamic expressions when the presentation time is brief.^17^ In addition, static emotional expressions activate motion-sensitive regions, as they imply biological motion.^26^ The pictures used are referred to as C, GS, JJ, MF, MO, NR, PF and WF in the dataset from Ekman and Freisen.^25^ An elliptic mask was fitted to reveal the face itself while hiding hair and ears (Fig. 1A). Each stimulus was presented 54 times in a random order. The duration of each stimulus was 500 ms, with a 500 ms interval between stimuli.

### Intracranial recordings: quantification and statistical analysis

#### Data processing

During the experiments, continuous SEEG was recorded with a 1000 Hz sampling rate by means of a 192 channel-EEG device (EEG-1200 Neurofax, Nihon Kohden^®^). The channels of interest were referred to a lead in a neutral white matter (neutral reference). A band-pass filter (0.015–300 Hz) was applied to avoid any aliasing effect. Each trial was epoched with a (200 ms, 700 ms) time window, with respect to the onset of the stimulus. Recordings were visually inspected by clinicians to ensure the absence of artefacts or epileptic activity. Channels with pathological EEG activity, when present, were discarded. Although this procedure cannot completely exclude the presence of interictal pathological high-frequency oscillations, not always detectable by visual inspection and potentially biomarkers of the epileptic zone,^27,28^ the fact that each region was sampled on different patients and different electrodes allowed us to make this possibility negligible.

Analyses were focused on iERPs that, compared to other types of SEEG signals (e.g. gamma-band power), are more sensitive and able to better identify modulations of both excitatory and inhibitory neural activity. IERPs were computed for all leads located in the grey matter, using a subtractive baseline correction applied versus the prestimulus interval (−200/0). Data analysis was carried out by using EEGlab toolbox (freely available at https://sccn.ucsd.edu/eeglab/index.php) to implement data segmentation, time-frequency decomposition and averaging across epochs.

#### Statistical analysis

A preliminary analysis was performed to identify responsive leads among the ones localized in the cortical grey matter. For each lead, we evaluated the significance of the iERPs elicited by each of the two experimental conditions (i.e. smiling and fearful expressions) after dividing each time window in 20 ms long time-bins following the stimulus onset, by applying a *t*-test to post-event bins versus pre-event baseline (α = 0.05). To decrease the false-positive ratio, only leads with significant responses in at least four consecutive time bins were designated as responsive. Considering that the responsiveness to the presented stimuli can be accounted for by both high-level aspects of face processing and low-level visual features of the stimulus, this result does not tell of the contribution of the responsive regions to the processing of emotional expressions. All leads showing a significant iERP in at least one experimental condition (smiling and fearful) were subsequently considered.

#### Discrimination between emotional and neutral expressions

To assess whether the response to each of the two emotional expressions (smiling and fearful) was significantly different from that elicited by neutral expressions, we performed two distinct two-way repeated measures ANOVAs using Condition (emotional, neutral) and Time (35 adjacent 20-ms time bins in the 0 ms, 700 ms window) as within-subject factors. For each lead showing a significant interaction and at least one significant main effect, *post hoc* analysis was conducted by means of a paired *t*-test according to a planned comparison design. A third two-way repeated measures ANOVA using the same criteria compared smiling and fearful faces, and was applied to all leads located in the frontal/insular regions and showing significant iERP in one of the two experimental conditions (smiling and fearful expressions). Data processing and statistical analysis were performed by using in-build functions implemented in MATLAB 2015a or EEGLAB.^29^

#### Continuous maps

To provide a continuous view of the topographic pattern of active leads, we built a circular mask based on the geodesic distance between two cortical points (i.e. the minimum pathway within the grey matter connecting the source and the target nodes). For each cortical node, we defined the nodes within a 1-cm geodesic distance from the original node and weighted the contribution of each node by a sigmoid function. Node weight was defined as a logistic function with unitary amplitude, a steepness of 2 and a midpoint at 7.5 mm. As a result, each node of the cortical mesh was associated with a collection of surrounding nodes, with all nodes within 5 mm of the origin maximally weighted, whereas those between 5 and 10 mm were gradually reduced in weight, to avoid edge effects. By this approach, we computed three different functional variables:^24,30,31^

- Cortical sampling density, i.e. the number of explored leads per cm^2^, using the fixed number (7) of nodes per lead and the average surface of a disk.
- Overall responsiveness, i.e. the number of responsive nodes as a percentage of the number of explored nodes within a disk. Given the fixed number of nodes representing a lead (7), this variable is equivalent to the number of responsive leads as a percentage of the number of explored leads within a disk. This variable provides an overall picture of the cortical responsiveness, ranging from 0% to 100%, directly comparable to neuroimaging studies. Data were thresholded at 10% to exclude the contribution of sparsely responsive regions.
- Relative responsiveness, i.e. the proportion of leads significantly selective to emotional expressions, of the overall number of responding leads. These maps provide a topographic picture of the frontal/insular selectivity of the emotional expressions. Data were thresholded at 10% to exclude the contribution of sparsely responsive regions.

#### Localization and region of interest analysis

The regional responsivity was assessed by (i) parcelling the entire cortical mantle according to the Lausanne2008 (resolution 60) template, which subdivides the entire brain into 129 different cortical and subcortical structures^32,33^ (Fig. 1); and (ii) estimating, for each region of the template, the percentage of leads showing a significant effect in the ANOVAs over the total number of investigated leads. The selection of this template was a compromise between different needs, as the more narrow-meshed a parcellation is, the more likely it is that effective connectivity data between two specific areas are missing. Frontal/insular regions showing a significant effect in at least 10% of the overall number of recording leads, evaluated on at least four leads, were selected for the study of the effective connectivity (see later). Results for the occipito-temporal network and parietal regions will not be discussed in depth due to our focus on motor/emotional resonances and because of their extensive treatment in previous human intracranial recordings investigations.^34-41^ Sampling and results relative to these regions are, however, reported in Supplementary Table 1.

### High-frequency electrical stimulation

After the recording of spontaneous seizures, HF-ES was performed through the electrodes in many cerebral structures, aimed at both inducing seizures and brain mapping. Bipolar HF-ES of pairs of adjacent leads was carried out by means of biphasic rectangular stimuli of alternating polarity (frequency: 50 Hz; pulse width: 0.5–1 ms; duration: 5 s, current intensity: up to 4 mA). Stimulations were delivered while patients were maintaining the Mingazzini position and speaking aloud, to evaluate upper limbs movements, speech arrest and other behavioural modifications. All the elicited responses were video-recorded and prospectively stored in clinical report documents. Here we collected the available results obtained by HF-ES of the frontal/insular sites showing a significant interaction between emotional (smiling or fearful) and neutral expressions. The aim of this analysis was to better characterize the functional role of the sites modulated by the visual stimulus, and it does not exclude that other responses can be evoked by HF-ES of other frontal/insular regions not activated by the visual task.

Based on our previous work,^42,43^ we characterized the type of clinical response according to the following categories: sensorimotor manifestation, interoceptive sensation, emotional behaviour, language impairments, unspecific subjective responses and unresponsive sites.

### Effective connectivity

The study of the effective connectivity was based on the open-source dataset Functional Brain Tractography Project f-tract (https://f-tract.eu/),^44,45^ which reports large-scale human brain connectivity maps based on CCEPs recorded in several hundreds of SEEG patients following SPES. The f-tract dataset is adapted to different atlas releases, including the Lausanne2008-60 atlas that we used to compute the regional responsivity in our study.

To study the internal connectivity of the frontal/insular regions involved in the processing of emotional expressions, where most of the responding leads were located, we focused on the data reporting the ‘afferent probability’ and the ‘efferent probability’, that is, the probability of afferent and efferent connectivity between the various regions of interest (ROIs) of the template. As described before, we considered only the regions showing a significant interaction in either positive versus neutral or negative versus neutral comparisons. For each ROI, a map of the afferent and the efferent probability was created. In addition, we displayed the matrix of probability across the regions of interest, and we grouped the distances among patterns of probability using a hierarchical binary cluster tree.

## Results

### Intracranial recordings

#### Frontal/insular responsiveness to emotional expressions

Recordings were obtained from 5663 recording leads [Left (L) = 2391; Right (R) = 3272] located in the cortical grey matter, with 2147 (L = 857; R = 1290) exploring the prefrontal, premotor and anterior insular cortices (see Fig. 1B for sampling density maps). Significant iERPs were elicited in ∼20% of these regions, during either smiling or fearful conditions (L = 176, R = 252 and L = 192, R = 215, respectively). Not surprisingly, the percentage of significant leads in these regions was lower than the overall percentage of significant leads recorded over the entire cortical sheet, i.e. including occipital and temporal regions (∼30%, i.e. L = 683, R = 1008 for smiling and L = 633, R = 993 for fearful expressions; Supplementary Fig. 1). Only leads located in prefrontal, premotor and anterior insular cortices showing significant responsiveness were subjected to further analyses.

#### Selectivity to smiling expressions

The two-way repeated measures ANOVA comparing smiling and neutral faces showed significant interaction, and at least one significant main effect, in 159 of 428 frontal/insular responding leads (L = 75/176; R = 84/252). A chi-squared test suggested the lack of significant lateralization (χ^2^ = *P* > 0.05). Interestingly, the percentage of significant leads in these frontal/insular regions (37.1%) was higher than the overall percentage of significant leads recorded over the entire cortical sheet, i.e. including occipital and temporal regions (25.5%; i.e. 427 of 1691 responding leads; see also Supplementary Table 1). Comparing these data with those obtained on the responsiveness (above), it can be deduced that, compared to occipital and temporal regions, frontal/insular regions have a higher selectivity for emotional faces, despite an overall lower responsiveness to the face stimulus.

**Figure 2:**
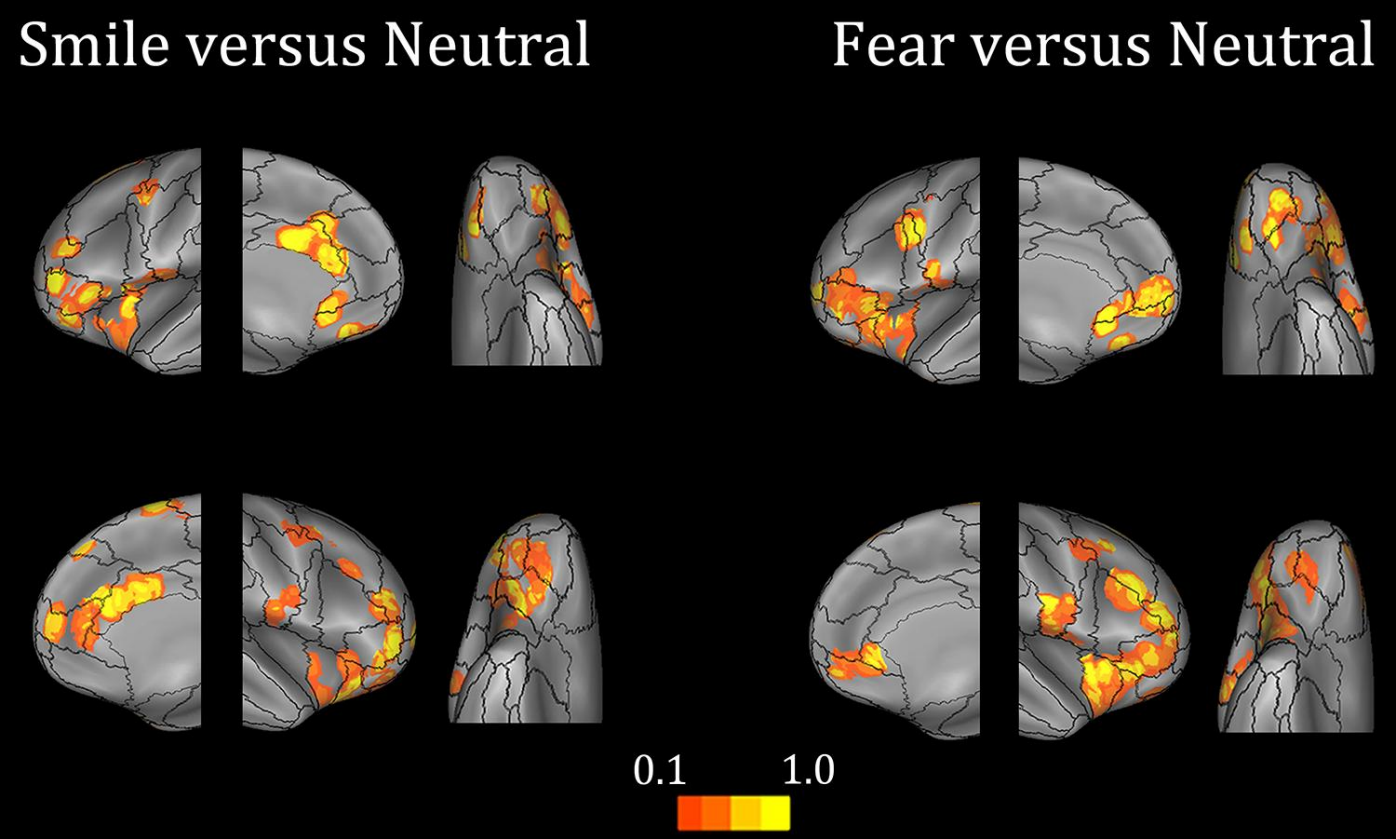
**Prefrontal, premotor and insular regions activated by emotional expressions**. Proportion of sites showing significant differences between emotional and neutral responses, of the overall number of the responsive sites, is plotted on the fs_LR brain template. The color scale indicates the percentage of responsive sites within a disk 1 cm in radius and centred on each node of the mesh.

The regional selectivity was assessed by parcelling the entire cortical mantle according to the Lausanne2008-60 template^32^ and estimating, for each region of the template, the percentage of leads showing a significant effect in the ANOVAs. Frontal/insular regions in which the significant number of leads is >10% of the recording leads include four territories: (i) the anterior cingulate region, where significant responses were bilaterally observed in the caudal anterior cingulate cortex (cACC), partially invading the territory of the rostral ACC (rACC) and in the adjacent ventromedial prefrontal cortex (vmPFC; labelled as ‘superior frontal 1’ in the Lausanne2008-60 template); (ii) the orbitofrontal region, that includes the lateral orbitofrontal cortex (OFC)1 and lateral OFC2 ROIs of the Lausanne2008-60 template, with a rightward lateralized reactivity; (iii) the dorsolateral prefrontal region, with significant effects in the left pars triangularis of the inferior frontal gyrus (IFG) and the bilateral anterior insula (AI), i.e. IFG-pt/AI henceforth; and finally, (iv) we found a high number of leads having significant reactivity in two premotor regions that, for reasons related to the borders of the parcellation provided by the Lausanne2008-60 template, failed to reach the 10% threshold: (a) in the bilateral Rolandic operculum (RO; located in the ventral part of the postcentral 3 region of the Lausanne2008-60) significant modulation was found only in the ∼6% of the leads, given the unresponsiveness of the leads located in the dorsal part of the same ROI; and (b) bilaterally in between the inferior frontal junction (IFJ), where the leads with significant modulation were distributed over three distinct ROIs of the Lausanne2008-60 parcellation (Fig. 2 and Table 1). Results relative to the occipito-temporal and parietal regions are reported in Supplementary Table 1.

**Table 1:** Regions responding to the observation of positive and negative expressions

	Smiling versus Neutral					Fearful versus Neutral				
	Right		Left		Lateral	Right		Left		Lateral
ROI	Resp. leads	%	Resp. leads	%	χ^2^	Resp. leads	%	Resp. leads	%	χ^2^
**ACC rostral**	4 (31)	**13%** ^a^	4 (18)	**22%** ^a^	*P* > 0.05	4 (31)	**13%** ^a^	5 (18)	**28%** ^a^	*P* > 0.05
**ACC caudal**	7 (44)	**16%** ^a^	8 (31)	**26%** ^a^	*P* > 0.05	2 (44)	5%	0 (31)	0%	–
**Superior frontal_1**	6 (61)	**10%** ^a^	2 (35)	6%	*P* > 0.05	1 (61)	2%	5 (35)	**14%** ^a^	***P* < 0.05** ^a^
Superior frontal_2	2 (68)	3%	2 (26)	8%	–	1 (68)	1%	0 (26)	0%	–
Superior frontal_3	1 (62)	2%	3 (30)	10%	–	3 (62)	5%	2 (30)	7%	–
Superior frontal_4	5 (96)	5%	2 (48)	4%	–	0 (96)	0%	0 (48)	0%	–
Frontal pole	0 (0)	0%	1 (7)	14%	–	0 (0)	0%	3 (7)	43%	–
**Lateral orbitofrontal_1**	9 (26)	**34%** ^a^	1 (35)	3%	***P* < 0.05** ^a^	0 (26)	0%	7 (35)	**20%** ^a^	***P* < 0.05** ^a^
**Lateral orbitofrontal_2**	5 (43)	**12%** ^a^	1 (29)	3%	*P* > 0.05	4 (43)	**9%** ^a^	6 (29)	**21%** ^a^	*P* > 0.05
Medial orbitofrontal	1 (63)	2%	3 (41)	7%	–	3 (63)	5%	4 (41)	**10%** ^a^	–
**Rostral middle frontal_1**	5 (63)	8%	4 (36)	**11%** ^a^	*P* > 0.05	6 (63)	**10%** ^a^	1 (36)	3%	–
Rostral middle frontal_2	1 (37)	3%	3 (12)	25%	–	1 (37)	3%	2 (12)	17%	–
Rostral middle frontal_3	1 (4)	25%	0 (13)	0%	–	0 (4)	0%	0 (13)	0%	–
Caudal middle frontal	2 (88)	2%	0 (62)	0%	–	5 (88)	6%	0 (62)	0%	–
IFG pars opercularis	2 (48)	4%	1 (46)	2%	–	4 (48)	8%	1 (46)	2%	–
IFG pars orbitalis	3 (36)	8%	3 (28)	11%	–	2 (36)	6%	0 (28)	0%	–
**IFG pars triangularis**	7 (104)	7%	12 (98)	**12%** ^a^	*P* > 0.05	16 (104)	**15%** ^a^	15 (99)	**15%** ^a^	*P* > 0.05
Precentral_1	1 (28)	4%	1 (14)	7%	–	2 (28)	7%	0 (14)	0%	–
Precentral_2	3 (40)	8%	1 (23)	4%	–	0 (40)	0%	1 (23)	4%	–
Precentral_3	1 (26)	4%	2 (16)	13%	–	2 (26)	8%	3 (16)	19%	–
Precentral_4	0 (87)	0%	2 (59)	3%	–	1 (87)	1%	5 (59)	8%	–
**Rolandic operculum**	7 (117)	6%	4 (60)	7%	–	17 (117)	**15%** ^a^	6 (60)	**10%** ^a^	*P* > 0.05
Postcentral_1	0 (6)	0%	0 (2)	0%	–	0 (6)	0%	0 (2)	0%	–
Postcentral_2	1 (26)	4%	1 (26)	4%	–	0 (26)	0%	1 (26)	4%	–
Paracentral lobule	1 (18)	6%	1 (15)	7%	–	0 (18)	0%	0 (15)	0%	–
**Insula (anterior)**	9 (100)	9%	14 (75)	**19%** ^a^	*P* > 0.05	18 (100)	**18%** ^a^	8 (75)	**11%** ^a^	*P* > 0.05
Insula (posterior)	4 (95)	4%	8 (91)	9%	–	6 (95)	6%	5 (91)	5%	–

The table illustrates, for each investigated prefrontal, premotor and anterior insular regions, the results of the comparison between emotional versus neutral expressions. The parcellation is based on the Lausanne2008 (resolution 60) atlas. For both smiling versus neutral and fear versus neutral comparison, we report the number of significant leads over the number of recording leads (indicated within brackets), the percentage and the results of the chi-squared test. ACC = anterior cingulate cortex; IFG = inferior frontal gyrus; Resp = responsive; ROI = region of interest.

^a^Regions showing a significant effect in at least 10% of the overall number of recording leads, evaluated on at least four leads, are indicated in bold.

#### Selectivity to fearful expressions

The two-way repeated measures ANOVA comparing fearful and neutral faces showed a significant interaction, and at least one significant main effect, in 167 of 407 frontal/insular responding leads (L = 74 of 192; R = 93 of 215). A chi-squared test assessed the absence of any lateralization (χ^2^ = *P* > 0.05). As in the case of smiling expressions, the percentage of significant leads in the frontal/insular regions (41.0%) was higher than the overall percentage of significant leads recorded over the entire cortical sheet (29.8%; i.e. 484 of 1626 responding leads), confirming a higher frontal/insular selectivity for fearful faces, despite an overall lower responsiveness to the face stimulus.

Frontal/insular regions showing a significant effect in at least 10% of the recording leads were similar to those identified by the previous analysis, with few but important differences: (i) in the anterior cingulate region, we found the bilateral reactivity of the rACC, extending to the adjacent left vmPFC (superior frontal 1 in the Lausanne2008-60 template), but we did not find any modulation in the cACC; (ii) in the OFC the modulation was significantly leftward lateralized, rather than rightward lateralized as it was in the smiling versus neutral comparison; (iii) in the AI/IFG region, significant modulation was similar to the case of the smiling versus neutral comparison; and (iv) in the premotor regions we found significant modulation in the RO (in which reactivity reaches the 10% threshold in the fearful versus neutral comparison), and in between the caudal middle frontal gyrus and the IFJ (as in the previous case, this reactivity was distributed over three distinct ROIs of the Lausanne2008-60 parcellation, none of which reach 10% of significant leads; Fig. 2 and Table 1). Results relative to the occipito-temporal and parietal regions are reported in Supplementary Table 1.

#### Timing of frontal/insular reactivity during the processing of emotional expressions

**Figure 3:**
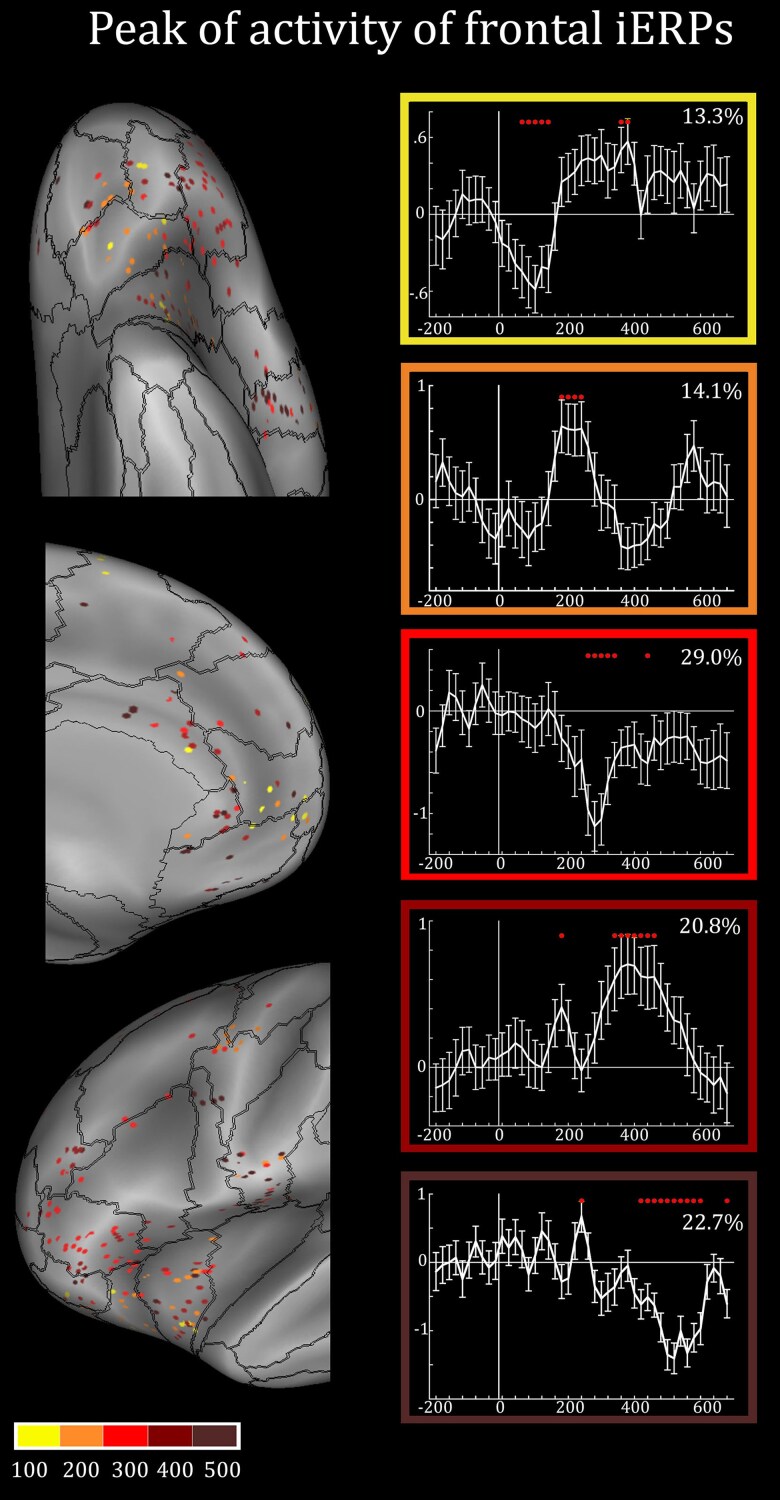
**Timing of intracranial event-related potential responses**. *Left*: The figure illustrates, for each site showing significant differences between emotional and neutral responses, the peak latency following stimulus onset. Both left and right contacts are plotted on the left hemisphere of the inflated surface of the FS_LR brain template. *Right*: Representative intracranial event-related potentials (iERPs) for each of the five peak latencies. Numbers within each frame indicates, for each peak latency, the corresponding percentage of sites.

For each frontal/insular lead showing a significant interaction, we identified the peak of the iERPs elicited by the observation of the smiling/fearful faces. Overall, we found peaks with latencies every 100 ms (±50), that is, at about 100 ms, 200 ms, 300 ms, 400 ms and 500 ms following stimulus onset (Fig. 3), with the majority of iERPs recorded from 300 ms onwards.

As shown in Fig. 3, the distribution of the iERP peaks on the cortical sheet does not reveal a sharp topography, although four interesting trends can be noted: (i) earliest peaks (100/200 ms) were more frequently recorded from the OFC and the vmPFC; (ii) peaks at 300 ms were predominant in the IFG-pt; (iii) late potentials (i.e. 400/500 ms) were mainly recorded from the RO and the ACC (rACC and cACC); and (iv) the AI exhibited a mixed pattern, characterized by the presence of both early and late iERPs.

### High-frequency electrical stimulation

Electrical stimulations (HF-ES; see the ‘Materials and methods’ section) were performed on 273 (L = 139; R = 134) sites over the 327 sites (L = 160; R = 167) showing a significant effect in the ANOVA comparing emotional (smiling or fearful) and neutral expressions. The remaining sites were not stimulated, coherently with the fact that HF-ES is typically performed by selecting only a few sites for each specific anatomical structure. All the frontal/insular regions of interest have been stimulated by HF-ES.

Behavioural responses or subjective manifestations were elicited in 103 leads (L = 54; R = 49), while the remaining 62% of leads were unresponsive to HF-ES.

**Figure 4:**
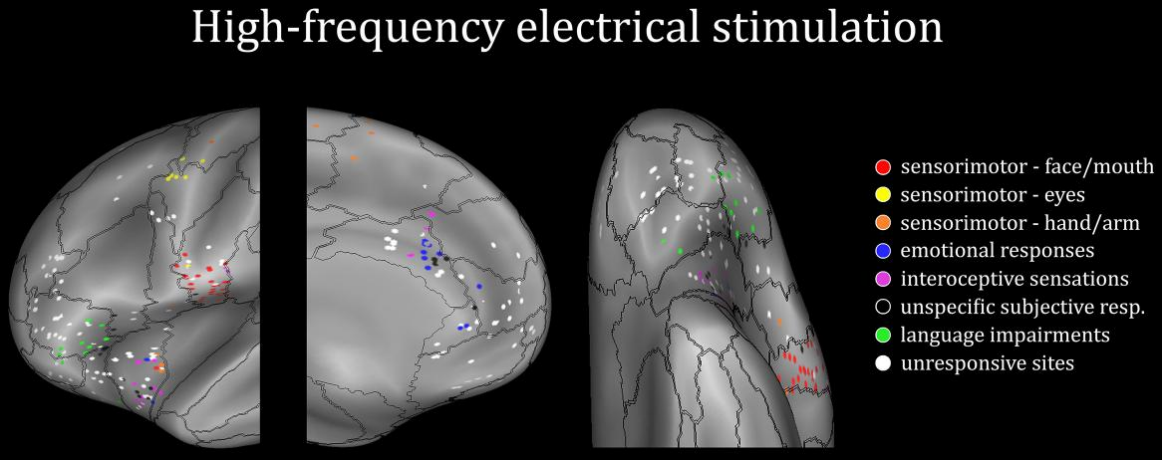
**Responses elicited by high-frequency electrical stimulation**. Anatomical distribution of the sites whose electrical stimulation elicits behavioural and subjective responses belonging to the seven categories of response, and unresponsive contacts. Both left and right contacts are plotted on the left hemisphere of the inflated surface of the FS_LR brain template.

Sensorimotor manifestations were found in 50 sites (L = 27; R = 23), almost exclusively located in RO and IFJ. These manifestations affected different body districts, including the face/mouth region (L = 18; R = 11), the eye/neck region (L = 5; R = 3) and hand/arm (L = 4; R = 9). The stimulation of the RO elicited effects limited to the face/mouth region, such as mouth movement inhibition, dysarthric speech, paresthetic sensations to the mouth region and, remarkably, facial movements including smiling expressions. The stimulation of the IFJ elicited eyelid clonicity and contralateral versive eye/neck movements. Finally, hand/arm atony and paraesthesia were more rarely elicited and scattered across different regions (Fig. 4).

Interoceptive manifestations, emotional responses and other undescribed subjective phenomena were found in 41 sites (L = 20; R = 21), almost exclusively from AI and cACC. Interoceptive manifestations (L = 7; R = 7) consisted of nausea and bodily sensations and were predominantly elicited from the AI and, to a lesser extent, from the cACC. Emotional responses (L = 7; R = 5) included positive and negative valence emotions. Positive emotions consisted of mirthful laughter and were elicited only from the cACC. Negative emotions included fear and anxiety and were elicited from the AI. Unspecific subjective manifestations (L = 6; R = 9) such as dizziness, sense of confusion and mental blunting were elicited from the AI and the cACC. AI has the lowest stimulation thresholds (Supplementary Fig. 2), although it gives the most negative responses to stimulation. This data excludes the hypothesis that the negative valence of the evoked response is merely attributable to greater stimulation intensities.

Language impairments including tachyphemia and speech arrest were found in 12 sites (L = 7; R = 5), almost exclusively from the ventral part of IFG-pt.

Unresponsive stimulations were found in 171 sites (L = 86; R = 85). They were frequently obtained in all investigated structures but predominant in the vmPFC, the OFC and the dorsal aspect of the IFG-pt. Most unresponsive regions were typically stimulated at 3 mA (Supplementary Fig. 2), while the majority of 1 mA stimulations were clustered in responsive ones (i.e. AI and RO). It follows that the lack of response in the former cannot be traced back to factors related to the stimulation parameters. For this reason, we rated these sites as unrelated, or at least less directly involved, in either motor or emotional/interoceptive functions.

### Effective connectivity

We extracted from the open-source dataset Functional Brain Tractography Project f-tract^44,45^ (https://f-tract.eu/) the ‘afferent and efferent probability values’ of all regions showing a significant interaction in at least 10% of the recording leads in either smiling versus neutral or fearful versus neutral comparisons, plus the precentral subfield where the majority of IFJ leads were located (‘precentral 3’ according to the Lausanne2008-60 template).

**Figure 5:**
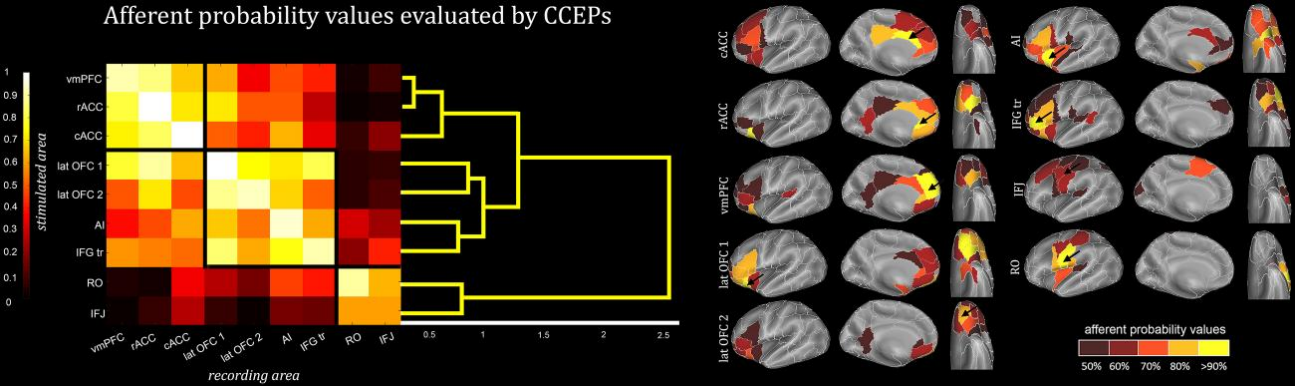
**Afferent effective connectivity assessed by cortico-cortical evoked potentials (CCEPs)**. *Left*: Matrix of the afferent connectivity probability across the regions showing significant differences between emotional and neutral responses. Data were based on the Functional Brain Tractography Project f-tract using the Lausanne2008 (resolutions 60) parcellation schema. Distances are grouped among patterns of probability using a hierarchical binary cluster tree. *Right*: Afferent connectivity probability (*n* ≥ 50%) estimated from CCEP data recorded following single-pulse electrical stimulation. The black arrow indicates the region from which CCEPs were recorded. AI = anterior insula; cACC = caudal anterior cingulate cortex; IFGtri = inferior frontal gyrus pars triangularis; IFJ = inferior frontal junction; Lat OFC1 = lateral orbitofrontal cortex 1; Lat OFC2 = lateral orbitofrontal cortex 2; rACC = rostral anterior cingulate cortex; RO = Rolandic operculum; vmPFC = ventromedial prefrontal cortex.

The resulting pattern of connectivity, depicted in Fig. 5A, gathers the analysed regions into four predominant clusters that match the above-described cingulate, orbitofrontal, dorsolateral prefrontal and premotor sets of regions. A first (cingulate) cluster encompasses the vmPFC and the rostral and caudate ACC, which represents an independent anatomical unit. A second (orbitofrontal) cluster is constituted by lateral OFC1 and OFC2, which is closely connected with a third (lateral prefrontal) cluster, grouping together the IFG-pt and the AI. A fourth (premotor) cluster is constituted by the RO and IFJ, corresponding to the ventral part of the postcentral_3 ROI and the precentral_3 ROI of the Lausanne2008-60 template, respectively. As illustrated in Supplementary Fig. 3A, the pattern of connectivity relative to the efferent probability values shows very similar segregation, supporting the hypothesis that these connections are predominantly bidirectional.

Figure 5B shows, for each region, the afferent connectivity with probability values >0.5. Three results deserve to be mentioned. First, the cingulate, the orbitofrontal and the lateral prefrontal clusters show dense reciprocal interconnections, while the premotor cluster appears to represent an island itself, poorly connected with all the prefrontal ones (values <40% in Fig. 5A). Second, among the regions of interest, the pattern of afferences of the premotor regions RO and IFJ is markedly different and predominantly dependent on the lateral parieto-frontal networks, rather than on prefrontal ones. Third, among the listed afferences, the only one involved in the visual processing of emotional expressions is the amygdala, projecting to both lateral OFC 1 and AI. Notably, all these observations also apply to the analysis of the efferent connectivity (Supplementary Fig. 3B).

## Discussion

In the present study, we investigated the contribution of prefrontal, premotor and anterior insular regions to the processing of emotional expressions, by combining intracranial recording, electrical stimulation and effective connectivity. A specific aim of the study was to disentangle between the ‘motor resonance’ model—hypothesizing that emotional expressions activate the observer motor/premotor cortices^7^—and the ‘emotional resonance’ model—stating that others’ expressions directly activate the emotional/interoceptive system, bypassing the motor system.^12^

Intracranial recording revealed widespread reactivity in four sectors, three of which are located in prefrontal/insular regions (ACC, OFC and AI/IFG) and one occupying premotor territories (RO and IFJ). While these data show that all these regions take part in the processing of others’ emotional expressions, the electrical stimulation of the same sites indicated that only some of them are directly involved in the production of emotional/interoceptive or motor responses. More specifically, HF-ES revealed that (i) in ACC and AI, the stimulation of the sites responding to emotional expressions elicited emotions and interoceptive sensations, coherently with the prediction of the ‘emotional resonance’ model; (ii) in RO, the stimulation of the sites responding to emotional expressions elicited face/mouth sensorimotor responses, including facial expressions, coherently with the ‘motor resonance’ model; and (iii) other activated regions (i.e. OFC, IFG-pt, IFJ) were largely unresponsive to HF-ES, or revealed functions unrelated to the processing of emotional expressions, suggesting that their recruitment during the passive observation task should be explained by reasons other than those attributable to any (either emotional or motor) resonance mechanism. The study of the effective connectivity showed that the prefrontal/insular sectors, which include ACC and AI where emotional/interoceptive responses were elicited, are reciprocally interconnected. In contrast, the premotor cluster—which includes RO, where face/mouth sensorimotor responses have been elicited—is part of the parieto-frontal circuits and poorly connected with the prefrontal/insular areas.

These results support the hypothesis that the passive observation of emotional expressions recruits (at least) two streams of information, directed toward independent emotional and motor territories, which differs in terms of functional fingerprints and connectivity. All these aspects are discussed in turn below.

### The anterior cingulate-insular system as a hub for ‘emotional resonance’

The ACC and the AI are the only regions that were both modulated by the observation of emotional expressions and reacted to HF-ES by eliciting emotional/interoceptive manifestations. These two results indicate that, in these regions, the visual information on other’s emotions is encoded by the same functional territories that contribute to the observer emotional state. The effective connectivity study revealed that the ACC and the AI are tightly interconnected, as also demonstrated by structural and functional connectivity data.^46,47^ Although these results indicate many functional affinities, the effect of the stimulation also reveals an interesting difference between the two regions. In the AI, the elicited effects were typically negative (e.g. fear, anxiety or nausea) while the stimulation of the cACC often elicited positive emotional behaviours (e.g. mirthful laughter).

The recruitment of the cACC during the observation of positive emotions is in line with previous evidence obtained by neuroimaging studies.^16,48-50^ Similarly, the evidence that the stimulation of the same territory elicits positive emotions and emotional behaviours was independently demonstrated by electrical stimulation studies.^21,43,51-55^ The discovery of a shared circuit for the perception and expression of positive emotions has been anticipated by two recent studies. First, in 2020 our group showed that the same ACC sites whose stimulation elicited mirthful laughter show an increase of the high-gamma band (50–150 Hz) during the observation of dynamic, but not static, expressions of happiness.^21^ The fact that, in the present study, also static expressions were able to elicit a response can be realistically attributed to the different type of signal analysed in the two studies, i.e. iERP versus high-gamma band. The differences between iERPs and high-gamma activity represents a crucial and still unsettled issue, but it is worth mentioning that previous intracranial studies have shown that visual stimuli—including faces—could modulate iERPs but not high-gamma activity in both high-order visual regions and non-visual cortical regions.^37,56,57^ Moreover, a new study demonstrated that 130 Hz continuous stimulation applied to the cACC biases the processing of static facial stimuli obtained by morphing happy, sad and neutral, leading participants to rate morphed faces as significantly happier during the stimulation of the cACC.^53^

Similar to the case of the ACC, the reactivity of the AI during the observation of positive and negative expressions is also in line with many neuroimaging^6,48,58-60^ and intracranial^61^ investigations. Concerning the electrical stimulation, previous studies demonstrated that, in line with our data, the HF-ES applied to the AI typically elicits unpleasant interoceptive sensations and negative emotions such as disgust and fear,^62-65^ albeit new data show that positive emotions can also be elicited from this region.^66^

Taken together, these data make the ACC and the AI the ideal neural substrate for an ‘emotional resonance’ system, that is, a system projecting the visual representation of other’s emotional displays into the corresponding emotional motor pathway, potentially facilitating facial mimicry and emotional contagion and possibly influencing the observer emotional state. The early timing of insular responses and its afferences from the amygdala, demonstrated by our effective connectivity data, makes the AI (along with the OFC) an ideal entry point of emotional visual information, also considering the early reactivity of the amygdala to emotional expressions.^67,68^ In contrast, the ACC is more likely involved in the behavioural response triggered by the observed emotional expression, that is, facial mimicry and emotional contagion.^14^ This hypothesis fits with a combined functional MRI-EMG study demonstrating that the spontaneous reaction to happy facial expressions, assessed by increased EMG activity in the zygomaticus major and orbicularis muscles, correlates with blood oxygen level-dependent activity in the ACC.^16^ Hence, also considering that the ACC direct projection to the facial nucleus,^69,70^ and its position at the top of the emotional motor pathway for facial expressions,^71^ it is tempting to speculate that the late timing of the iERPs recorded from the ACC (300–500 ms) could represent a motor potential elicited by the automatic facial mimicry, which is indeed typically recorded at the peripheral level at the same time window.^9^

### The Rolandic operculum and its contribution to ‘motor resonance’

The RO differs from all the other areas activated by our stimuli for being the only region where HF-ES elicited face/mouth sensorimotor responses, including facial expressions. This result is fully in line with previous stimulation data from our and other groups.^72,73^ Its contribution to the control of volitional facial movements is substantiated by the evidence that its lesion is associated to impaired voluntary (but not spontaneous) movements of the lower face^74-76^ and that emotional facial expressions elicited by the stimulation of the RO are typically devoid of emotional content.^21,73^ The quintessential sensorimotor nature of this region is also confirmed by its connectivity with ventral premotor, primary motor and parietal regions, which clearly reveals the belonging of this area to the parieto-frontal circuits involved in the control of goal-related actions,^3,77^ as well as by its projections to the facial motor nuclei throughout the internal capsule.^70^

The recruitment of this region during the observation of emotional expressions has been occasionally observed also by neuroimaging investigations.^78,79^ Interestingly, a recent study investigated the shared circuits for facial emotion production and observation, reporting that this region was activated during the observation and execution of positive (happy) and negative (angry) expressions.^80^ Additional insights for the role of the RO in the processing of facial expressions come from lesion studies, reporting that RO lesions impair the explicit recognition of both positive and negative emotional expressions.^81^

Our study shows that the visual input reaches this region very late, never before 300 ms and usually at about 500 ms. As in the case of the ACC, such a late response is compatible with a motor potential evoked by the observed expression. Being facial mimicry recorded at the peripheral level at about 500 ms following the stimulus presentation,^9^ there must be at least one motor region active in a period compatible with such response—and the data described above make the RO, along with the ACC, the ideal candidate.

All these data pave the way for the hypothesis that emotional expressions trigger two parallel systems. One—controlled by the ACC-AI circuit—associates the observed expression to one’s own emotional/interoceptive representations, as predicted by the ‘emotional resonance’ model. The other—centred on the RO—superimposes the visual information on other’s expression on the observer motor representation, as hypothesized by the ‘motor resonance’ model. Both systems react very late to the observed stimulus and, at least in principle, both of them are in the position to explain the phenomenon of automatic facial mimicry.

### Non-mirror regions responding to emotional expressions

The OFC, the pars triangularis of the IFG and the IFJ were activated by the passive observation of emotional expressions, coherently with the face-selective prefrontal patches described in the macaque.^82,83^ However, all these regions were largely unresponsive to HF-ES, or revealed functions unrelated to the processing of emotional expressions, indicating that the recruitment of these regions during our passive observation task should be explained by reasons other than those attributable to any emotional or motor resonance mechanism.

#### Orbitofrontal cortex as a potential entry point for emotional information

The OFC was activated by emotional expressions, in line with previous intracranial recordings showing that the primate OFC contains visual neurons reflecting face expression and face identity.^84,85^ Intracranial ERPs from the OFC were very early (100/200 ms), confirming in humans the same short latencies observed in the macaque OFC using single-neuron recordings.^85^ The study of its effective connectivity highlighted, besides a tight connection with both ACC and AI, afferences from the amygdala. However, the OFC was unresponsive to the electrical stimulation, showing that this region does not play a predominant role in initiating emotional behaviours or goal-directed actions directly. The lateralization of smiling (right) and fearful (left) expressions is an unexpected finding, possibly due to the rightward and leftward lateralization of rewarding stimuli and fear extinction processes, respectively.^86,87^ However, SEEG lateralization effects must be taken with a grain of salt as the majority of an inter-hemispheric difference derive from different groups of patients, thus adding on top of the investigated effects the inter-individual variability.^88^

Taken together, this set of evidence points to the OFC as a potential entry point of the information directed toward the emotional fields of ACC and AI. This interpretation is compatible with the shared view^89^ that the OFC gathers sensory information about the stimulus identity from the amygdala, evaluates its reward value and, on the basis of its rewarding/non-rewarding nature, guide behavioural choices by modulating other frontal regions including ACC and AI.^90-92^ Hence we hypothesize that the predominant source of information feeding the ‘emotional resonance’ system is not necessarily mediated by input from the temporal lobe, as in the case of the mirror-neuron system for action recognition, but rather by subcortical routes encompassing the superior colliculus-pulvinar-amygdala pathway,^93-98^ eventually reaching the anterior cingulate-insular system through the OFC.

#### Inferior frontal gyrus pars triangularis and inferior frontal junction are not part of the mirror-neuron system

The modulation of the IFG-pt and the IFJ during the processing of emotional expressions is typically interpreted as a sign of mirror-neuron activation.^16,80,99-101^ The results obtained by electrically stimulating these regions, and their effective connectivity, suggest taking this assumption with a grain of salt.

Concerning the IFG-pt, its effective connectivity is markedly different from what can be expected from a human homologue of the Macaque’s ventral premotor cortex, where mirror neurons have been originally described.^102^ Indeed, IFG-pt connections are mainly with prefrontal territories, rather than with parieto-frontal circuits as it is in the case of the Macaque mirror-neuron system.^103^ Most important, the electrical stimulation of this region gave no response, or produced language impairments, showing that the IFG-pt is not involved in the production of facial actions or in the generation of emotional responses.

Concerning the IFJ, this region is close and partially overlapping with the dorsal premotor cortex, commonly conceived as part of the action observation network.^104-106^ While its effective connectivity confirms its belonging to the parieto-frontal circuits hosting the mirror-neuron system, the stimulation of this territory elicited contralateral versive eye/neck movements and eyelid clonicity, making a strong case for the role of this region in the control of gaze/neck movements driven by attention-demanding stimuli. Remarkably, previous studies showed that the IFJ response to faces appears to be driven primarily by the eyes.^107^

Starting from the consideration that emotional expressions are a specific class of highly salient stimuli, one may expect that their observation should activate systems involved in reorienting attention when behaviourally relevant targets are detected. Hence, a possible interpretation is that IFG-pt and IFJ may, respectively, take part to the ‘ventral attentional systems’, specialized for the detection of behaviourally relevant stimuli, and the ‘dorsal attentional system’, involved in top-down orienting of attention.^108-111^ In line with this interpretation, these regions are usually activated during the explicit recognition of emotional facial expressions, rather than their passive views.^18^

### Two mirror systems in the frontal lobe?

Fifteen years ago, proponents of the so-called ‘simulation theory’ argued that others’ emotional expressions trigger in the observer an automatic inner simulation and, at a behavioural level, the automatic facial mimicry of the expression.^4,5^ The proponents of the simulation theory also provided a limited number of mutually exclusive explanatory models, two of which are still considered by system neuroscientists. The ‘motor resonance’ model assumes that perceiving other’s emotions triggers a multistep serial processing starting with the simulation of the expression in the perceiver's motor system, where facial movements are represented, and continuing with the sorting of this information to brain regions involved in social cognitive functions.^7,8,112^ The ‘emotional resonance’ model, in contrast, hypothesizes that perceiving emotional displays directly activates the corresponding emotional regions, such as the ACC and the AI,^12^ bypassing premotor/motor regions.

Although they were often thought of as mutually exclusive models, the present data suggest that the two systems coexist, and that emotional displays simultaneously activate two partially independent networks, served by different streams of information and potentially involved in different functions.

The ‘emotional resonance’ system, anchored to the anterior cingulate-insular network, receives broad and coarse visual information from the OFC, which in turn is the recipient of input from the superior colliculus-pulvinar-amygdala pathway.^89,93,98^ Once projected to the ACC and AI, the visual input activates specific emotional/interoceptive representations. While the identification of its specific functions is of course beyond the potentialities of our study, our data are compatible with the hypothesis that this system may serve socio-emotional functions, such as the facilitation of emotional contagion, spontaneous mimicry and social synchronization.^14,113^ This interpretation is compatible with the evidence that lesions of the ACC and OFC impair not only the recognition of others’ emotions,^114,115^ but also the production of overt socio-emotional displays, deficits in social interactions and changes in social behaviour.^116-118^

The ‘motor resonance’ system, hosted by the RO, is in contrast part of the parieto-frontal circuits and represents the homologue of the classic mirror-neuron system for hand actions. The RO is not directly connected to any temporal region endowed with visual properties, hence it is likely that—as in the case of the mirror-neuron system for hand actions—the visual input reaches the RO from inferior parietal regions, that are in turn recipient of action-related visual information from superior and middle temporal regions.^119^ Following the evidence that lesions of the RO impair the explicit recognition of emotional expressions,^81^ one may speculate that this system contributes to action recognition strategies by exploiting one’s own motor representations as a heuristic strategy to understand others’ communicative intents.

The debate on motor and emotional resonance is inextricably linked to the debate on facial mimicry.^9,10^ Whether facial mimicry reflects an automatic motor response, or an emotional one, is still unsettled and this issue is in fact the focus of very recent studies.^11^ From a system neuroscience perspective, it has often been assumed that this automatic facial action could be controlled by the ventral part of the voluntary motor system, where facial movements are represented,^7,8^ but new data point at a wider network of cortical and subcortical regions.^16^ Even if we did not record EMG on our patients, our stimuli were the very same as Ekman and Friesen’s^25^ set of pictures of facial affect used by Dimberg^9^ in his seminal studies. It is therefore reasonable to assume that the participants of our study were also producing below threshold facial changes. Capitalizing on the late iERPs recorded in the ACC and the RO, on the result of their stimulation, revealing the occasional production of emotional facial expressions, and their well-known descending projections to the facial nuclei,^69,120^ we hypothesize that these regions are the best candidates for transforming observed expressions into produced expressions. If on the one hand, this consideration allows us to greatly limit the list of areas potentially involved in the genesis of this phenomenon, on the other hand, it does not still clarify whether facial mimicry is the output of motor resonance (via RO) or emotional resonance (via ACC), with all that follows in terms of its contribution to action recognition or social synchronization.

In conclusion, the present study contributes to a longstanding interdisciplinary debate on the simulation mechanisms active during face-to-face social interactions. Capitalizing on the results of intracranial recordings, electrical stimulation and effective connectivity studies, it suggests that the two predominant models in the literature on mirror neuron—namely, the emotional resonance and the motor resonance models—are not mutually exclusive. Rather, they coexist and work in parallel, but they differ in terms of neural bases (ACC-AI versus RO), visual pathways (amygdala-orbitofrontal versus temporo-parietal), content (emotional/interoceptive versus motor representations) and functions (socio-emotional synchronization versus action recognition).

## Supplementary Material

awae050_Supplementary_Data

## Data Availability

The conditions of our ethics approval do not permit public archiving of individual anonymized raw data. Readers seeking access to the data should contact the corresponding author. Access will be granted to named individuals in accordance with ethical procedures governing the reuse of sensitive data. Specifically, requestors must sign a formal agreement confirming that (i) the user may not use the database for any non-academic purpose; (ii) the document must be signed by a person with a permanent position at an academic institute or publicly funded research institute. Up to five other researchers affiliated with the same institute for whom the signee is responsible may be named at the end of this document which will allow them to work with this dataset; and (iii) the user may not distribute the database or portions thereof in any way.
