# Supplementary material for: Anatomo-functional basis of emotional and motor resonance elicited by facial expressions
Source: Brain. 2024 Feb 14;147(9):3018–31. doi: 10.1093/brain/awae050 (PMC12007602; doi:10.1093/brain/awae050)
Supplement: awae050_Supplementary_Data [file awae050_supplementary_data.pdf]

Table S1. Occipito-temporal and parietal regions responding to the observation of positive and negative expressions.

The table illustrates the results of the comparison between emotional vs neutral expressions in occipito-temporal and parietal regions. The table is based on the parcellation provided by the Lausanne2008 (resolution 60) atlas and covers all the posterior regions, not reported in Table 1. For both smiling vs neutral and fear vs neutral comparison, we report the number of significant leads over the number of recording leads (indicated within brackets), the percentage and the results of the chi-square test. Regions showing a significant effect in at least 10% of the overall number of recording leads, evaluated on at least 4 leads, are indicated in bold.

	SMILING VS. NEUTRAL					FEARFUL VS. NEUTRAL				
	Right		Left		Lateral.	Right		Left		Lateral.
ROI	Resp. leads	%	Resp. leads	%	Chi2	Resp. leads	%	Resp. leads	%	Chi2
amygdala	2 (17)	12%	7 (22)	**32%***	p>0.05	6 (17)	**35%***	5 (22)	**23%***	p>0.05
inferior parietal_1	2 (69)	3%	3 (28)	11%	---	5 (69)	7%	0 (28)	0%	---
inferior parietal_2	1 (41)	2%	2 (23)	9%	---	2 (41)	5%	1 (23)	4%	---
supramarginal_1	0 (47)	0%	2 (54)	4%	---	0 (47)	0%	2 (54)	4%	---
supramarginal_2	6 (56)	**11%***	5 (81)	6%	p>0.05	0 (56)	0%	5 (81)	6%	---
posterior cingulate_1	2 (61)	3%	1 (41)	2%	---	4 (61)	7%	2 (41)	5%	---
superior parietal_1	0 (21)	0%	1 (19)	5%	---	1 (21)	5%	0 (19)	0%	---
superior parietal_2	3 (15)	20%	0 (5)	0%	---	2 (15)	13%	1 (5)	20%	---
superior parietal_3	3 (37)	8%	0 (3)	0%	---	3 (37)	8%	1 (3)	33%	---
temporal pole	0 (11)	0%	0 (17)	0%	---	0 (11)	0%	0 (17)	0%	---
cuneus	2 (27)	7%	0 (2)	0%	---	0 (27)	0%	0 (2)	0%	---
precuneus_1	2 (44)	5%	0 (11)	0%	---	1 (44)	2%	0 (11)	0%	---
precuneus_2	3 (76)	4%	3 (20)	15%	---	6 (76)	8%	1 (20)	5%	---
entorhinal	3 (25)	12%	5 (36)	**14%***	p>0.05	2 (25)	8%	4 (36)	**11%***	p>0.05
hippocampus	5 (194)	3%	1 (63)	2%	---	15 (194)	8%	3 (63)	5%	---
parahippocampal_1	3 (19)	16%	3 (46)	7%	---	1 (19)	5%	7 (46)	**15%***	p>0.05
superior temporal_1	0 (35)	0%	5 (46)	**11%***	p>0.05	6 (35)	**17%***	2 (46)	4%	---
superior temporal_2	3 (125)	2%	13 (163)	8%	---	11 (125)	9%	6 (163)	4%	---
superior temporal sulcus	0 (16)	0%	3 (38)	8%	---	0 (16)	0%	4 (38)	**11%***	p>0.05
middle temporal_1	5 (53)	9%	0 (26)	0%	---	2 (53)	4%	1 (26)	4%	---
middle temporal_2	11 (134)	8%	14 (96)	**15%***	p>0.05	15 (134)	**11%***	0 (96)	0%	---
inferior temporal_1	5 (87)	6%	5 (126)	4%	---	6 (87)	7%	10 (126)	8%	---
inferior temporal_2	7 (95)	7	5 (80)	6%	---	11 (95)	**12%***	7 (80)	9%	---
lingual_1	7 (24)	**29%***	4 (9)	**44%***	p>0.05	6 (24)	**25%***	4 (9)	**44%***	p>0.05
lingual_2	11 (55)	**20%***	8 (21)	**38%***	p>0.05	7 (55)	**13%***	4 (21)	**19%***	p>0.05
fusiform_1	6 (55)	**11%***	3 (34)	9%	---	10 (55)	**18%***	7 (34)	**21%***	p>0.05
fusiform_2	11 (67)	**16%***	9 (71)	**13%***	p>0.05	17 (67)	**25%***	21 (71)	**30%***	p>0.05
isthmus cingulate	7 (50)	**14%***	1 (38)	3%	---	6 (50)	**12%***	1 (38)	3%	---
lateral occipital_1	11 (40)	**28%***	4 (10)	**40%***	p>0.05	9 (40)	**23%***	5 (10)	**50%***	p>0.05
lateral occipital_2	12 (73)	**16%***	1 (26)	4%	---	11 (73)	**15%***	3 (26)	12%	p>0.05
pericalcarine cortex	4 (53)	8%	1 (8)	13%	---	15 (53)	**28%***	1 (8)	13%	p>0.05

Figure S1. Responsiveness maps.

Proportion of responsive sites during the observation of positive (left) and negative (right) emotional expressions, out of the overall number of the recording sites. The color scale indicates the percentage of responsive sites within a disk 1 cm in radius and centered on each node of the mesh.

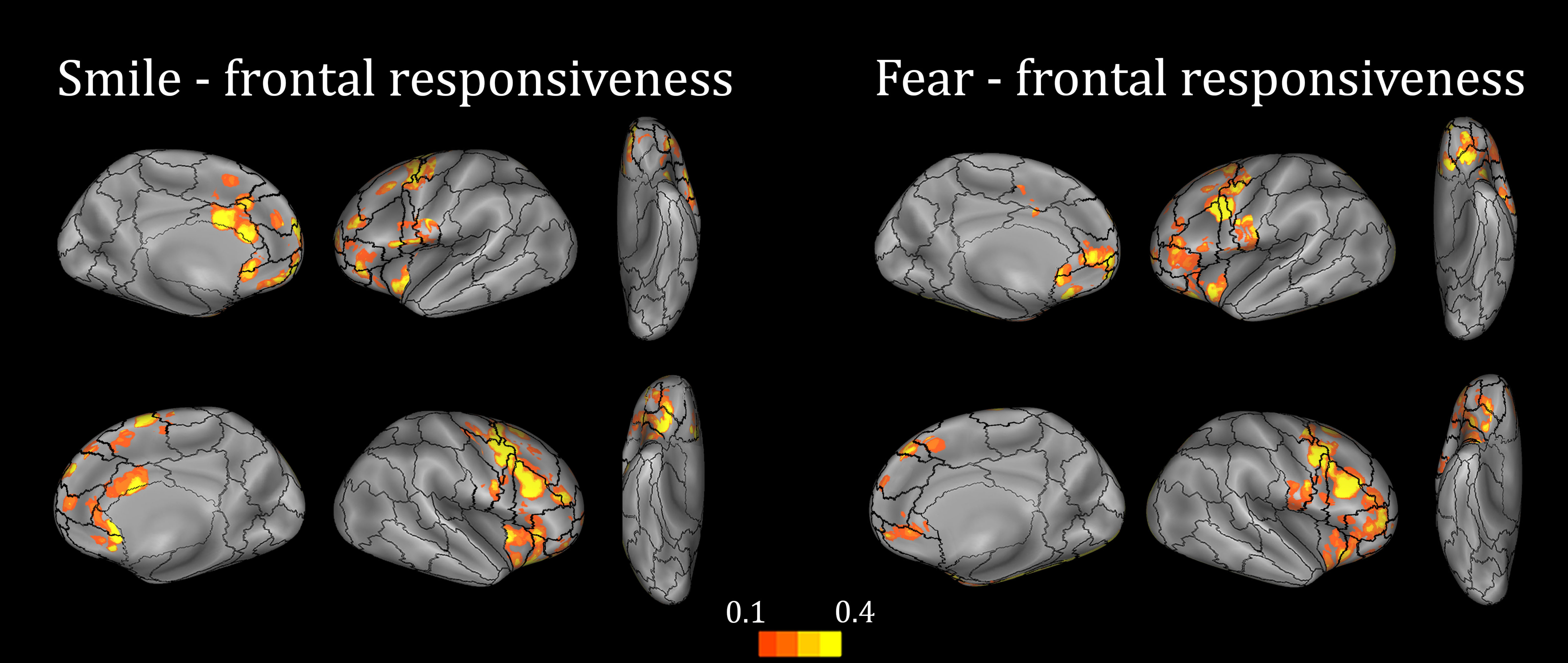
Smile - frontal responsiveness
Fear - frontal responsiveness
0.1
0.4

Figure S2. Responses elicited by high-frequency electrical stimulation.

The figure illustrates, for each stimulated site, the intensity with which the elicited behavioral and subjective responses were obtained. Both left and right contacts are plotted on the left hemisphere of the inflated surface of the FS_LR brain template.

High-frequency electrical stimulation (thresholds)

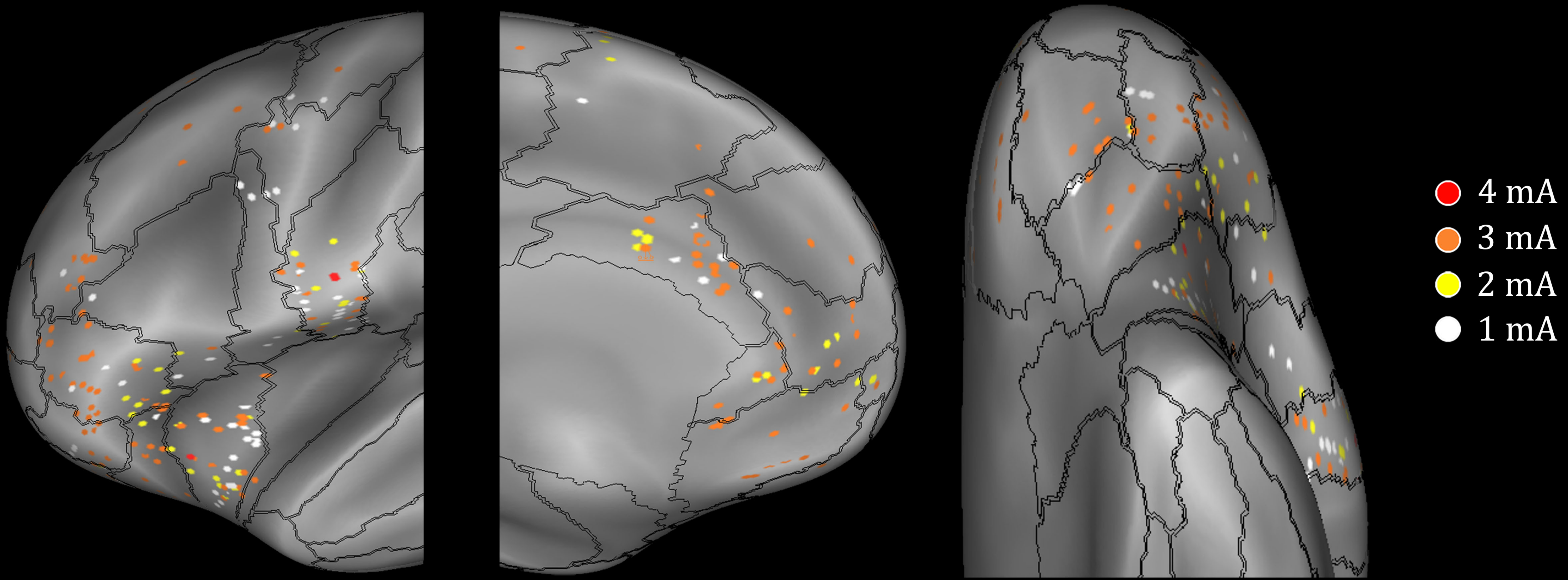

Figure S3. Efferent effective connectivity assessed by CCEPs.

Left panel. Matrix of the efferent connectivity probability across the regions showing significant differences between emotional and neutral responses. Data were based on the Functional Brain Tractography Project f-tract using the Lausanne2008 (resolutions 60) parcellation schema. Distances are grouped among patterns of probability using a hierarchical binary cluster tree. Right panel. Efferent connectivity probability (n≥50%) estimated from CCEPs data recorded following SPES. The black arrow indicates the region from which CCEPs were recorded.

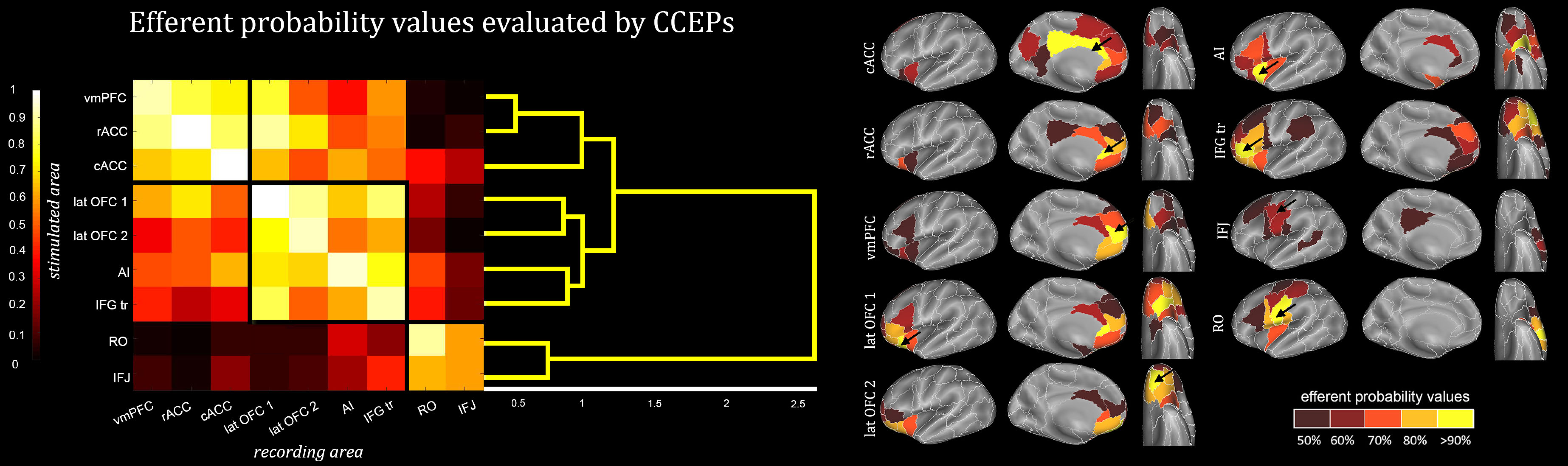

Efferent probability values evaluated by CCEPs
stimulated area
recording area
vmPFC
rACC
cACC
lat OFC 1
lat OFC 2
AI
IFG tr
RO
IFJ
1
0.9
0.8
0.7
0.6
0.5
0.4
0.3
0.2
0.1
0
0.5
1
1.5
2
2.5
efferent probability values
50%
60%
70%
80%
>90%
